# Multicentre outbreak of *Candida viswanathii* bloodstream infections linked to contaminated milrinone, Italy, April to October 2025

**DOI:** 10.2807/1560-7917.ES.2026.31.36.2600311

**Published:** 2026-09-10

**Authors:** Alessio Mesini, Giuseppe Spiga, Marta Ciofi Degli Atti, Debora Contu, Carmen D’Amore, Elisabetta Ugolotti, Gianluca Vrenna, Marco Castignone, Venere Cortazzo, Marco Merli, Lara Ricotta, Elisa Matarazzo, Elena De Carolis, Elisa De Pasqual, Maurizio Sanguinetti, Chiara Vismara, Paola Bernaschi, Anna Marchese, Roberto Bandettini, Carlo Federico Perno, Raffaele Spiazzi, Massimiliano Raponi, Elio Castagnola, Vincenzo Di Pilato, Candida viswanathii outbreak study group, Candida viswanathii outbreak study group, Erica Ricci, Marcello Mariani, Carolina Saffioti, Elisa Tavella, Emanuela Caci, Paola Barabino, Roberto Formigari, Guido Michielon, Andrea Moscatelli, Andrea Dato, Valeria Fox, Nadia Mores, Giuseppe Vetrugno, Giovanni Vento, Eleonora Nardi, Cecilia Del Curto, Marta Vecchi, Giovanna Travi, Edward Willison, Giancarlo Icardi, Andrea Orsi

**Affiliations:** 1 Infectious Diseases Unit, Department of Pediatrics, IRCCS Istituto Giannina Gaslini, Genoa, Italy; 2 Clinical Governance Unit, Health Management Department, IRCCS Istituto Giannina Gaslini, Genoa, Italy; 3 Epidemiology, Clinical Pathways and Clinical Risk Unit, Medical Direction, IRCCS Bambino Gesù Children's Hospital, Rome, Italy; 4 Laboratory of Microbiology, IRCCS Istituto Giannina Gaslini, Genova, Italy; 5 Microbiology and Diagnostic Immunology Unit, IRCCS Bambino Gesù Children's Hospital, Rome, Italy; 6 Pharmacy Unit, IRCCS Istituto Giannina Gaslini, Genoa, Italy; 7 Division of Infectious Diseases, ASST Grande Ospedale, Milan, Italy; 8 Complex Unit of Clinical Microbiology. Department of Laboratories, Azienda Socio-Sanitaria Territoriale (ASST) Grande Ospedale Metropolitano (GOM) Niguarda, Milan, Italy; 9 Dipartimento di Scienze di Laboratorio ed Ematologiche, Fondazione Policlinico Universitario A. Gemelli IRCCS, Rome, Italy; 10 SC Farmacia, ASST Grande Ospedale Metropolitano Niguarda, Milan, Italy; 11 Department of Surgical Sciences and Integrated Diagnostics (DISC), University of Genoa, Genoa, Italy; 12 UO Microbiologia, IRCCS Azienda Ospedaliera Metropolitana, Genoa, Italy; 13 Medical Directorate, IRCCS Istituto Giannina Gaslini, Genoa, Italy; 14 Medical Direction, IRCCS Bambino Gesù Children's Hospital, Rome, Italy; 15 The members of the group are listed under Collaborators

**Keywords:** *Candida viswanathii*, Candidaemia, Outbreak investigation, Contaminated medicinal product, drug safety surveillance

## Abstract

In 2025, a multicentre outbreak of bloodstream infections caused by *Candida viswanathii*, a rare non-albicans *Candida* species, was identified in Italy. The source was recognised after four *C. viswanathii* bloodstream infections were identified within a short period at a single centre. Initial local investigations did not identify environmental or patient-to-patient transmission. Given the strong geographical association of *C. viswanathii* with the Indian subcontinent, a hypothesis-driven investigation focusing on medicinal products of Indian origin was undertaken. Milrinone was the only common exposure among affected patients, and culture of the implicated product yielded *C. viswanathii*, confirming contamination. In total, 40 cases of *C. viswanathii* candidaemia were identified across four centres between April and October 2025; all were bloodstream infections. Most were associated with one predominant milrinone lot, although additional lots were implicated. Immediate control measures included withdrawal of the implicated product, notification of national authorities and active microbiological surveillance of exposed patients. This outbreak highlights the importance of clinician-led outbreak detection, hypothesis-driven epidemiology and pharmacovigilance in identifying rare, medicine-associated fungal infections.

**Key public health message**

**What did you want to address in this study and why?**

We investigated an unexpected cluster of rare *Candida viswanathii* bloodstream infections occurring in multiple Italian hospitals. Because this species is extremely uncommon in Europe, identifying the source was essential to prevent further cases and protect vulnerable patients. The investigation was also prompted by the increasing use of imported medicines related to limited availability of certain drugs from manufacturers in Europe.

**What have we learnt from this study?**

We found that the infections were linked to a contaminated medicinal product rather than to local hospital transmission. Rigorous epidemiological reasoning combined with targeted microbiological testing of the implicated drug and genomic characterisation of clinical and drug-derived isolates were essential to source identification. Diagnostic challenges and initial misidentification probably contributed to a delayed recognition of the outbreak.

**What are the implications of your findings for public health?**

Globalised pharmaceutical supply chains, driven in part by limited economic incentives for local production of some essential medicines, can introduce new infectious risks. Strong drug safety monitoring, accurate laboratory identification and rapid information sharing across centres are essential to detect and control medicine-associated outbreaks.

## Background

Since its description by Viswanathan and Randhawa in 1959 [1], *Candida viswanathii* has been reported only rarely as a human pathogen, with fewer than 40 cases described worldwide, mainly from the Indian subcontinent and only sporadically elsewhere; in Europe, only two cases have been reported, both from Spain [2]. Its apparent rare detection, however, probably reflects in part the difficulty of accurate species-level identification rather than true absence, so that the true incidence is likely to be underestimated. Despite its rarity, it has been associated with a wide spectrum of clinical manifestations, ranging from bloodstream infections to those of the central nervous system, as well as joint infections [2]. Conventional identification methods may misidentify *C. viswanathii* as closely related species, most notably *Candida tropicalis*, which has overlapping phenotypic and genetic characteristics [3]. *Candida viswanathii* is phylogenetically closely related to *C. tropicalis*, both cluster within the core *Candida* clade that also contains *C. albicans* and *C. parapsilosis* (the CTG clade). Although many species historically assigned to *Candida* have recently been reclassified into other genera of the Saccharomycotina [4], *C. viswanathii* itself is currently retained within the genus *Candida*.

Clinical management of *C. viswanathii* infections is complicated by the scarcity of published data and the absence of standardised treatment recommendations. Case reports describe successful management with prolonged courses of azole antifungals, particularly voriconazole and fluconazole, sometimes requiring chronic suppressive therapy in cases of recurrence or refractory infection. Surgical intervention, including debridement and washout, is frequently necessary in deep-seated infections [2]. Antifungal susceptibility testing revealed variable resistance patterns; some isolates exhibit elevated minimum inhibitory concentrations (MICs) to fluconazole, highlighting the importance of species-level identification and susceptibility-guided therapy [5].

From an epidemiological perspective, *C. viswanathii* infections have been reported in both community and healthcare-associated settings. Molecular typing studies have identified multiple clusters, suggesting the potential for outbreaks and/or common environmental or exogenous sources [5]. Although the pathogenicity of *C. viswanathii* is less well characterised than that of more common *Candida* species, it shares recognised virulence traits such as biofilm formation and adaptability to host environments [6].

Microbial contamination of medicinal products represents a well-recognised but infrequent cause of severe healthcare-associated outbreaks. Such events have been linked to invasive infections including fungal meningitis, endophthalmitis, septic arthritis and bloodstream infections, particularly following administration of contaminated injectable or compounded pharmaceuticals [7-10]. Immunocompromised patients and those undergoing invasive procedures are at highest risk for morbidity and mortality.

In addition to direct infectious risk, microbial contamination can alter the physicochemical properties of pharmaceutical products, leading to reduced efficacy or adverse reactions [11]. Several outbreaks associated with contaminated medications have resulted in fatalities and long-term sequelae, most notably the 2012 to 2013 outbreak of *Exserohilum rostratum* meningitis linked to contaminated methylprednisolone acetate [8,12]. Bacterial contamination, including outbreaks caused by intrinsically resistant organisms such as *Burkholderia cepacia* complex, has similarly resulted in nosocomial transmission and significant therapeutic challenges [13].

Despite regulatory oversight and lot recall mechanisms, lapses in good manufacturing practice (GMP), insufficient environmental monitoring and breaches in aseptic technique continue to pose risks [9,12,14]. Preventive strategies rely on strict adherence to GMP, robust environmental surveillance and rapid diagnostic methods to ensure early detection of contamination [7,12,14].

## Outbreak detection

In April 2025, the microbiology laboratory of the Istituto di Ricovero e Cura a Carattere Scientifico (IRCCS) Ospedale Pediatrico Bambino Gesù (OPBG) in Rome, Italy, identified *C. viswanathii* from a blood culture of a patient admitted to the intensive care unit (ICU) after cardiac surgery. Early isolates were initially misidentified as *Candida tropicalis* [3]. Over the period April to August 2025, additional cases occurred in patients with different diagnoses hospitalised on various wards. Infection prevention and control actions were strengthened, and extensive epidemiological, microbiological and environmental investigation conducted. All active screening tests for patient colonisation and environmental samples resulted negative. 

Subsequently, on 12 August 2025, the microbiology laboratory of the IRCCS Istituto Giannina Gaslini (IGG), a third-level paediatric hospital in Genoa, Italy, reported the identification of *C. viswanathii*, isolated from a blood culture of a patient admitted to the ICU after cardiac surgery. This event did not initially raise concern, as the implementation of updated diagnostic system libraries in recent years has allowed the identification of an increasing number of species compared with the past. A few days later, a second case was reported, essentially involving the same type of patient, this episode triggered an internal outbreak investigation at IGG. Since the patients had attended both the operating room and the ICU, environmental epidemiological investigations were carried out in both the cardiac surgery unit and the ICU, in addition to screening swabs from the two patients (skin sampling from armpits and groin, and rectal swabs), who tested negative. None of the investigations performed at that time yielded positive results, neither environmental cultures nor colonisation screening.

The hypothesis that the patients might have acquired the infection in the operating room remained plausible until a third and fourth case of *C. viswanathii* candidaemia were diagnosed at IGG. Unlike the previous cases, the third patient was admitted to the ICU and underwent diagnostic cardiac catheterisation but not cardiac surgery. This was the first case involving a non-operated cardiac patient. This case represented a break from the uniformity of the previous ones and called into question the epidemiological hypotheses that had been formulated up to that point. The investigators therefore considered which elements might be common among the various cases and given the strong epidemiological link between *C. viswanathii* and the Indian subcontinent, they explored whether the affected patients had received therapies or had implanted devices originating from that region. The only common factor identified was treatment with milrinone, a positive inotrope and vasodilator, and a selective phosphodiesterase III (PDE3) inhibitor used in paediatric cardiac surgery for reducing the incidence of low cardiac output syndrome [15,16]. Several vials of the drug were cultured, and on 4 October 2025, growth of *C. viswanathii* was detected.

We report a multicentre outbreak of *C. viswanathii* bloodstream infections in Italy associated with a contaminated drug, illustrating the epidemiological and diagnostic challenges posed by rare pathogens and the public health implications of contaminated medicinal products.

## Methods

### Study design

We conducted a multicentre outbreak investigation with active case finding. Following the alert, issued on 4 October, each participating centre searched its laboratory information system for blood cultures positive for *C. viswanathii*. In addition, one centre retrospectively reviewed all stored bloodstream isolates previously identified as *C. tropicalis* since 2023 to identify possible misidentified *C. viswanathii* isolates, while another centre confirmed the identity of its outbreak isolates by internal transcribed spacer (ITS) sequencing. Patients exposed to the implicated milrinone at the time of the alert also underwent prospective surveillance blood cultures.

### Data sources

Following notification of the contamination by IGG, each participating centre queried its laboratory information system to identify all episodes of *C. viswanathii* bloodstream infection. Given the extreme rarity of the pathogen, no temporal limits were applied to case finding; moreover, it was not possible to determine in advance how many drug lots were affected by the contamination. An ad hoc case report form (CRF) was developed on the REDCap [17,18] platform to build a fully anonymised database including all identified cases.

### Case definition

An episode was defined as the culture-based isolation of *C. viswanathii* from an otherwise sterile site in a patient treated with milrinone. Repeated episodes occurring within 15 days in the same patient were excluded from the analysis. No age limits were applied. Case finding was applied without temporal restriction owing to the rarity of the organism; all identified cases occurred between April and October 2025.

### Epidemiological and environmental investigation

All centres involved in the outbreak assessment performed epidemiological investigations. Patients with invasive *C. viswanathii* infection were tested with surveillance cultures to determine whether they were also colonised by *C. viswanathii*. Two of the four centres performed environmental sampling in areas visited by the patients. One centre tested bed-neighbours of infected patients for potential colonisation. All patient and environmental screening samples yielded negative results.

### Microbiological investigations

Blood culture samples were incubated in BACT/ALERT 3D (B.25 version, bioMérieux, France) or Bactec FX (Beckton Dickinson, United States (US)) instruments in aerobic/anaerobic bottles (bioMérieux; Beckton Dickinson) with an automated monitoring system.

Once the drug involvement was suspected, milrinone lactate was inoculated in aerobic bottles (bioMérieux; Beckton Dickinson); the positive sample was identified as a yeast by Gram stain and then sub-cultured (35 ± 2 °C overnight) on blood, chocolate and selective chromogenic media including Candida ID 2 (bioMérieux), COLOREX Candida Plus (Biolife Italiana, Italy), Chromatic Candida (Liofilchem, Italy) and CHROMagar Candida (Beckton Dickinson) agar plates for downstream analyses (see below).

Macroscopic growth characteristics of *C. viswanathii* and *C. tropicalis* were evaluated on four commercially available chromogenic media.

Yeast identification was performed either by MALDI-TOF MS (version 3 database), Vitek MS Prime (BioMérieux) or MBT Smart MALDI-TOF MS or MALDI Biotyper sirius (Bruker Daltonics, Bremen, Germany). All fungal isolates were stored at −80 °C in brain heart infusion broth (BHI) (Oxoid, United Kingdom) containing 20% v/v glycerol (Sigma Aldrich, US). The glycerol stocks were used as sources of isolates for all the experiments performed in this study. From frozen stocks, isolates were plated on Sabouraud Dextrose plates (Oxoid; bioMérieux) and/or selective chromogenic media, followed by overnight incubated at 37 °C before downstream analyses (i.e. genomic DNA extraction, antifungal susceptibility testing).

Antifungal susceptibility testing (AFST) was done by broth microdilution using Sensititre YeastOne ITAMYUCC antifungal susceptibility panel (Thermo Fisher Scientific, US) according to the Clinical and Laboratory Standards Institute (CLSI). We recorded MICs following 24/48 h incubation at 35 ± 2 °C. Interpretation of AFST results was not possible as no specific breakpoints are yet established for *C. viswanathii*. We used the CLSI-based Sensititre YeastOne panel because it is in routine use in the participating Italian laboratories and, in the absence of species-specific clinical breakpoints in either the CLSI or the European Committee on Antimicrobial Susceptibility Testing (EUCAST) system, it allows direct comparison of MICs with the existing *C. viswanathii* literature, which has been generated predominantly with CLSI methodology [2,5].

Total genomic DNA was extracted from fungal cultures as previously described [3,19]. Shotgun libraries were prepared from purified genomic DNA samples and sequenced with the NovaSeq 6000 or NextSeq 550 platforms using a 2 × 150 bp paired-end approach. Raw reads were assembled using SKESA v2.4.0 (https://github.com/ncbi/SKESA). Global phylogenomics was investigated by generation of core-genome single nucleotide polymorphisms (cgSNPs), using BWA v0.7.19 to align Illumina reads against *C. viswanathii* MIL-IGG (reference genome), the strain isolated from a contaminated batch of milrinone at IGG (BioSample no. SAMN53543980) [20]; RepeatMasker v4.2.1 (https://github.com/Dfam-consortium/RepeatMasker) and FreeBayes v1.1.0 (https://github.com/freebayes/freebayes) were employed to identify repetitive regions and call SNPs, respectively. The publicly available genomes of *C. viswanathii* ATCC 20962 (GCF_003327735.1) and NRRL Y-6660 (GCA_030566835.1) were included as outliers of the same species. Maximum-likelihood phylogenetic trees were constructed on concatenated cgSNPs by IQ-TREE v3.0.1, using a general time reversible (GTR) nucleotide substitution model [21]. 

## Results

### Geographical and temporal distribution of identified cases

The 40 cases comprised 27 male and 13 female patients. The affected population was predominantly paediatric (31/40 aged < 18 years, including 17 infants aged < 1 year) but spanned a wide age range (median: 2.1 years; range: 4 days to 67 years). Underlying conditions were operated cardiac disease in 24 patients, non-operated cardiac disease in eight, and non-cardiac conditions in eight. All 40 episodes were bloodstream infections, with no isolation from other sterile sites.

Following the notification of the milrinone lot contamination by IGG on 4 October, three additional Italian clinical centres responded: the IRCCS Ospedale Pediatrico Bambino Gesù (OPBG), the Fondazione Policlinico Universitario Agostino Gemelli, IRCCS (FPG), both based in Rome, and the Grande Ospedale Metropolitano Niguarda (NIG) in Milan. A total of 40 cases of *C. viswanathii* bloodstream infection were identified between April and October 2025. These cases were distributed as follows: 21 at OPBG, 10 at IGG, eight at NIG and one at FPG. The first cases identified at OPBG and NIG had occurred before those detected at IGG, over the period April to August 2025. The FPG reported a single case of *C. viswanathii* candidaemia; investigations on previously collected samples diagnosed as *C. tropicalis* bloodstream infections did not reveal any species misidentification by MALDI-TOF mass spectrometry systems, further confirmed by sequencing of the internal transcribed spacer (ITS). Similarly, at NIG all *C. tropicalis* isolates recovered from blood cultures since 2023 were retrospectively re-examined, without identification of *C. viswanathii*.

A preliminary, single-centre report describing part of this outbreak (15 of the 21 cases identified at OPBG) has been published [3]; the present study provides a complete, multicentre characterisation of the outbreak and identifies its common source.

**Figure 1:**
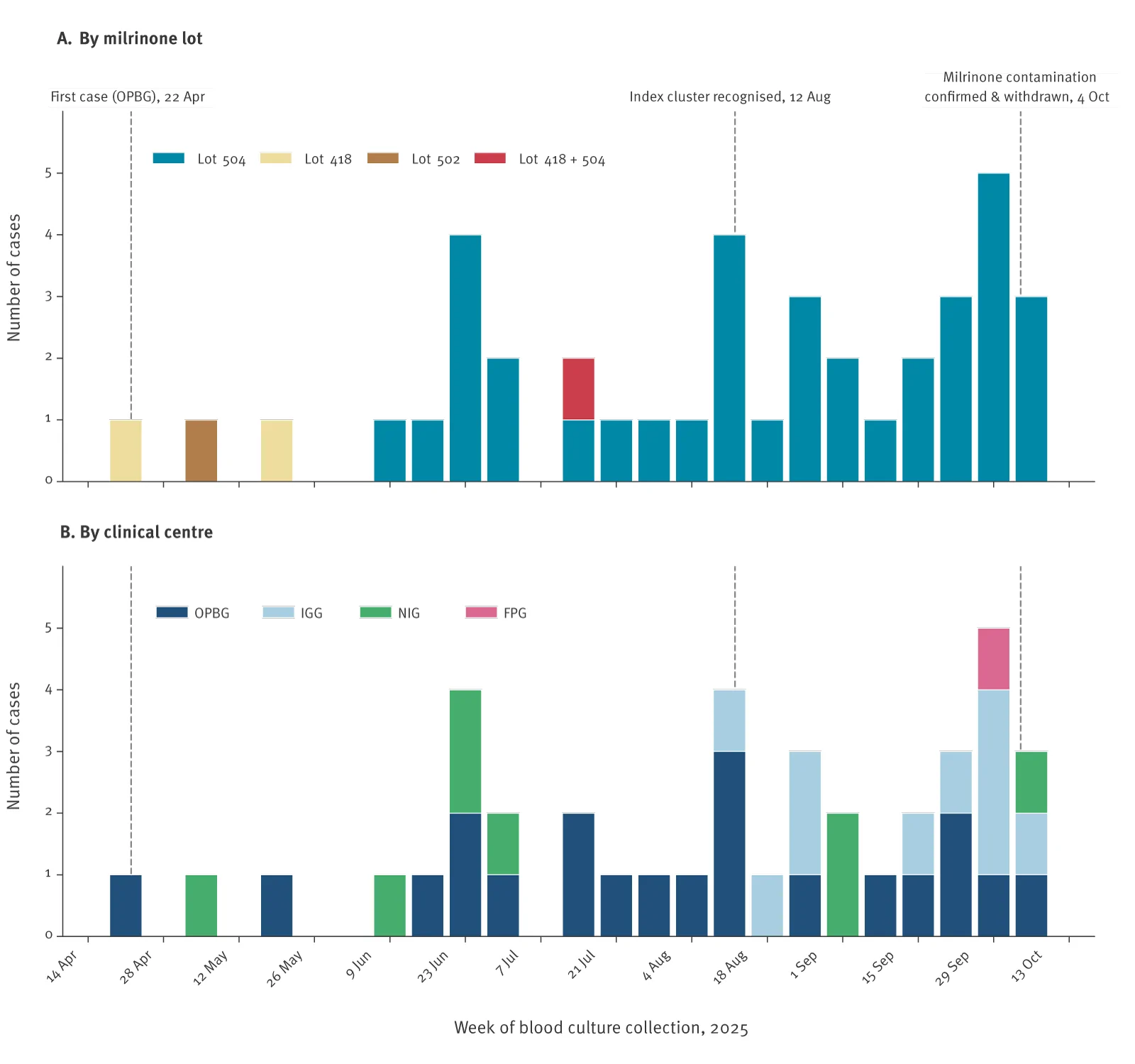
*Candida viswanathii* bloodstream infections by week, milrinone lot and centre, Italy, April–October 2025 (n = 40)

FPG: Fondazione Policlinico Universitario Agostino Gemelli, IRCCS; IGG: IRCCS Istituto Giannina Gaslini; IRCCS: Istituto di Ricovero e Cura a Carattere Scientifico; NIG: Grande Ospedale Metropolitano Niguarda; OPBG: IRCCS Ospedale Paediatrico Bambino Gesù.

Cases occurred over 8 months (from April to October 2025), with an increase observed during August and September 2025. The temporal distribution was compatible with repeated exposure to a common source rather than point-source transmission within a single healthcare setting. An epidemic curve and timeline of key events are shown in Figure 1.

### Drug exposure and lot involvement

All cases had documented exposure to milrinone. Three milrinone lots were implicated overall (lots 504, 418 and 502). One lot (number 504) accounted for most cases across centres (36/40); the remaining cases were associated with lots 418 (two cases) and 502 (one case), with one further patient exposed to both lots 418 and 504. This pattern indicates contamination at the manufacturing stage rather than an isolated single-lot event. Patient exposure to the drug are summarised in Table 1.

**Table 1:** Distribution of cases and milrinone-exposed patients by centre, Italy, April–October 2025 (n = 40)

Centre	Patients exposed to milrinone (n)	Cases (n)
OPBG	247	21
IGG	37	10
NIG	NA^a^	8
FPG	4	1

FPG: Fondazione Policlinico Universitario Agostino Gemelli, IRCCS; IGG: IRCCS Istituto Giannina Gaslini; IRCCS: Istituto di Ricovero e Cura a Carattere Scientifico; NIG: Grande Ospedale Metropolitano Niguarda; OPBG, IRCCS Ospedale Paediatrico Bambino Gesù.

^a^ Denominator data on patients exposed to milrinone were not available for NIG.

### Phenotypic characterisation of outbreak isolates

Results from AFST revealed that all tested isolates (n = 40) exhibited uniformly low MIC values for voriconazole, isavuconazole, caspofungin, anidulafungin, micafungin and amphotericin B, but higher MIC values for fluconazole (Table 2).

**Table 2:** In vitro antifungal susceptibility profiles of *Candida viswanathii* isolates characterised in this study, Italy, April–October 2025 (n = 40)

Antifungal agent	MIC range	MIC_50_	MIC_90_
Fluconazole	2–8	4	4
Voriconazole	0.125–0.5	0.25	0.5
Isavuconazole^a^	0.06–0.5	0.5	0.5
Caspofungin	0.06–0.5	0.125	0.5
Anidulafungin	0.06–0.25	0.125	0.125
Micafungin	0.03–0.125	0.06	0.06
Amphotericin B	0.125–1	0.25	0.5

MIC: minimum inhibitory concentration.

^a^ Data available for 32 of 40 isolates.

MIC range, MIC_50,_ MIC_90_ values reported as mg/L.

**Figure 2:**
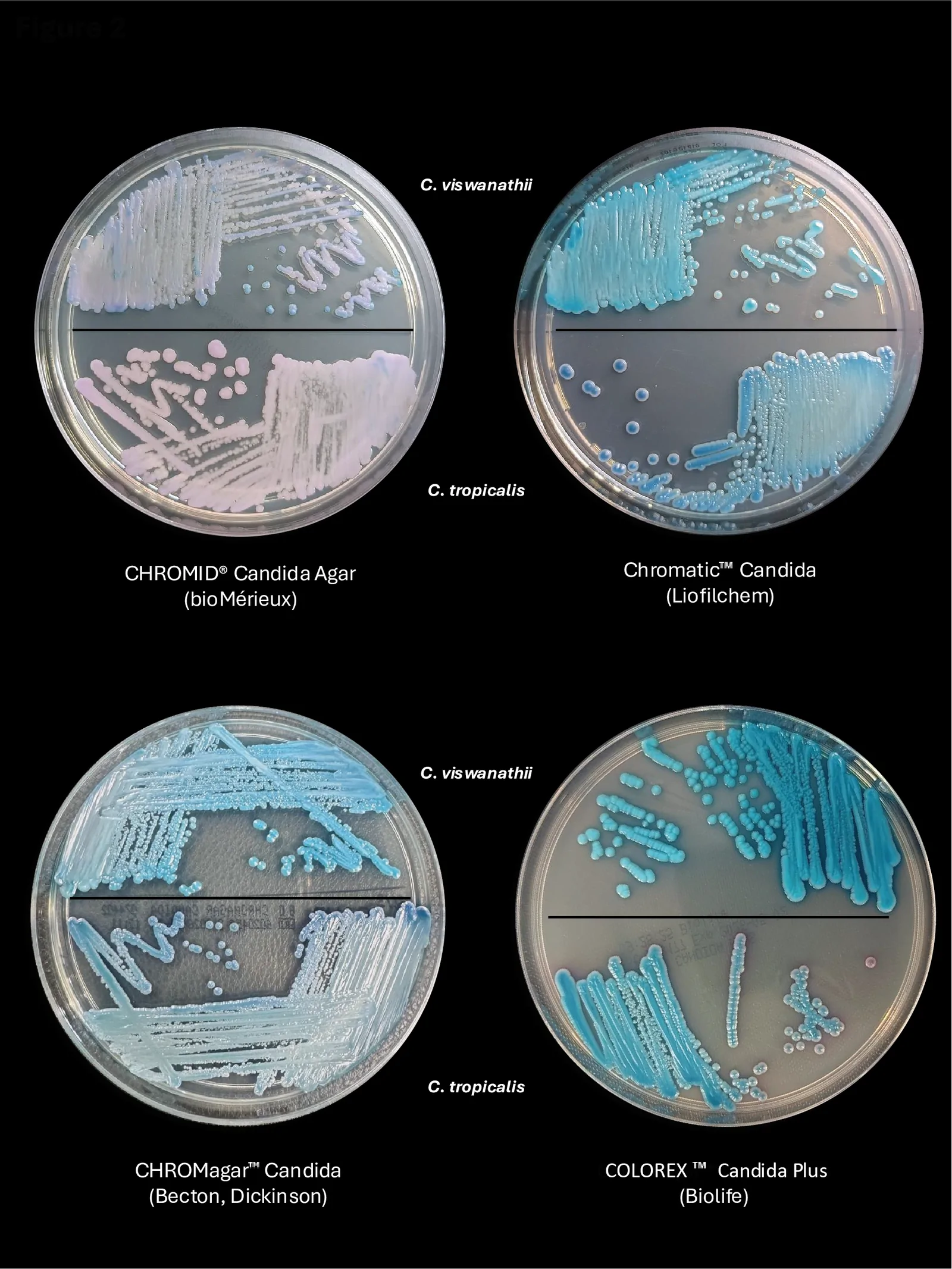
Comparative macroscopic morphology of *Candida viswanathii* and *Candida tropicalis* cultured on four chromogenic media from different manufacturers, Italy, April–October 2025

We observed clear differences in colony pigmentation and saturation across evaluated media and between species (Figure 2).

Colony morphology and chromogenic appearance of Candida viswanathii and Candida tropicalis on four commercial chromogenic media. Representative plates show *C. viswanathii* (upper half of each plate) and *C. tropicalis* (lower half) following incubation at 30 °C for 48 h. Colony texture and morphology were largely conserved between the two species, whereas pigmentation and colour saturation varied according to the medium. CHROMID® Candida Agar provided the clearest visual discrimination: *C. viswanathii* formed pale bluish-grey to lilac colonies, while *C. tropicalis* produced pink-to-beige colonies. On the other three media, the species differed primarily in the intensity and saturation of their blue-to-turquoise pigmentation; however, chromogenic expression was heterogeneous and differentiation was less reliable. Horizontal lines indicate the boundary between the two inoculated species on each plate.

Across the four chromogenic media, colony texture and morphology were largely conserved between species, whereas chromogenic expression varied markedly and was medium-dependent. CHROMID Candida Agar provided the most reliable visual discrimination, with *C. viswanathii* forming pale bluish-grey to lilac colonies vs the pink-to-beige colonies of *C. tropicalis*; on the other media the two species differed mainly in colour saturation and showed heterogeneous, less reliable differentiation (Figure 2).

### Genomic characterisation of outbreak isolates

**Figure 3:**
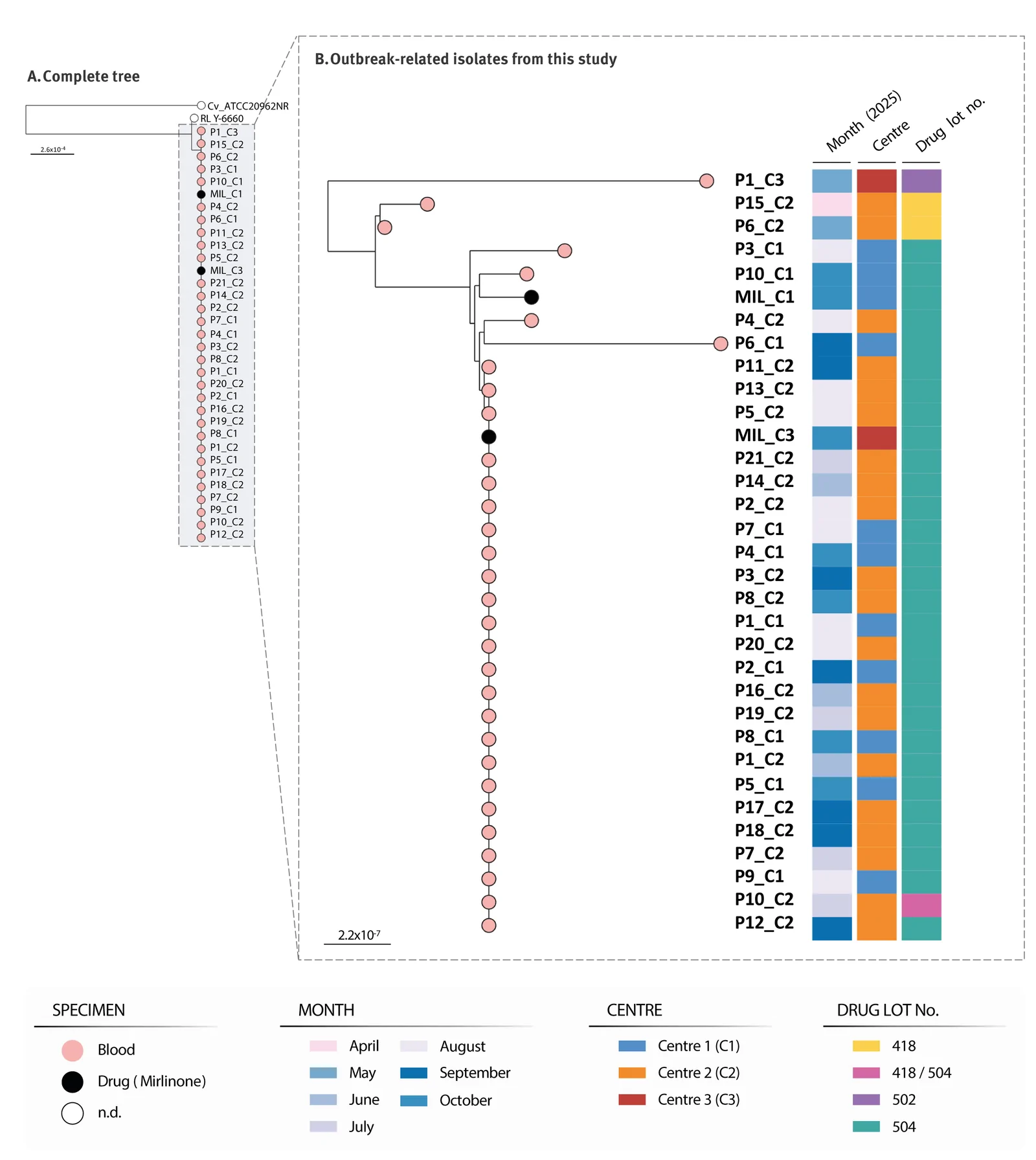
Maximum likelihood phylogeny of *Candida viswanathii*, Italy, April–October 2025 (n = 33)

Overall, 31 of 40 isolates of *C. viswanathii* recovered from blood at IGG, OPBG and NIG as well as two of three isolates recovered from drug vials (batch number 504) were subjected to whole genome sequencing (WGS) to assess clonal relatedness. According to phylogenetic analysis, all clinical isolates from Italian centres were resolved into a single tree branch, further including the two isolates cultivated from the contaminated drug vials, suggesting a high genomic relatedness in terms of separating cgSNPs (Figure 3A). Notably, isolates clustered according to the contaminated drug batch from which they originated. All isolates linked to batch 504 formed a monophyletic clade, whereas isolates linked to batches 418 and 502 clustered in distinct branches (Figure 3B). Investigation of cgSNPs confirmed a limited number of genomic differences separating the clinical and the drug isolates from the three Italian centres (cgSNPs min: 0–max: 16; median: 0; interquartile range (IQR): 0–2), pointing to a clonal origin. Consistently, comparative genomic analysis between outbreak-associated isolates and two epidemiologically unrelated *C. viswanathii* strains with available genome sequences (i.e. ATCC 20962 and NRRL Y-6660) revealed substantial genetic divergence, with a large number of separating cgSNPs (min: 563–max: 16,343; median: 16,018; IQR: 565–16,338). As no species-specific threshold for clonality is established for *C. viswanathii*, we assessed genomic relatedness from the distribution of pairwise cgSNP distances relative to epidemiologically unrelated control strains; the clear separation between the within-outbreak distances (0–16 cgSNPs) and those to the unrelated reference strains (> 563 cgSNPs) supports a clonal origin.

Included are isolates cultured from blood samples or contaminated drug vials, and epidemiologically unrelated reference strains included for comparative purposes. Tip colours indicate the specimen source. Additional metadata, including the date of isolation, source hospital and batch number of the contaminated drug vials, are provided.

### Outbreak control measures

Considering that IGG had supplied several vials of the drug to other hospitals in Italy, in addition to notifying the competent authorities, other clinical centres were also informed about the contamination.

After contamination of milrinone was confirmed on 4 October 2025, the implicated product was immediately withdrawn and replaced with alternative therapy. National health authorities and the Italian Medicines Agency were notified without delay. Clinical centres known to have received milrinone from IGG and from NIG were contacted directly. Information was subsequently shared more broadly at national level through professional healthcare networks to ensure rapid awareness.

The implicated medicinal product did not have a marketing authorisation in Italy and had been imported under the national procedure for foreign medicinal products. The hospital pharmacy notified the Italian Medicines Agency, which indicated that, for such a foreign medicinal product imported under the national regulatory framework, the competent authority is the Office for Maritime, Air and Border Health (USMAF), responsible for authorising the importation of foreign medicines; we therefore submitted the notification to USMAF and also informed the Italian wholesaler that had supplied the product. We did not make direct contact with the manufacturer or with the regulatory authorities in the country of origin, as this lies within the remit of the competent regulatory authorities.

Microbiological testing was performed on the only lot of the implicated product for which contamination had been suspected and which was physically available at the reporting centre; samples of the other lots were no longer available for testing. Milrinone was subsequently replaced by a different product obtained from a different importer and manufacturer, and a formal manufacturer-level trace-back of additional lots was outside the remit of the investigating centres.

At IGG, all patients still receiving milrinone or recently exposed at the time of the alert underwent active microbiological surveillance consisting of two blood cultures per day for 7 days. This strategy enabled the early detection of one asymptomatic candidaemia (the last case for IGG), allowing prompt antifungal treatment.

Similarly, at NIG, three patients receiving milrinone at the time of the alert were monitored with two sets of blood cultures every other day. One asymptomatic patient was found to have candidaemia 4 days after milrinone discontinuation and received antifungal therapy accordingly.

At OPBG, infection prevention and control measures implemented since the first cases included patient isolation and cohorting, reinforcement of contact precautions and hand hygiene, review of central line care practices, and increased environmental cleaning in patient care areas.

Following the alert about the contaminated milrinone lot, FPG sent an urgent circular for complete recall and prohibition of use of the lot, with prompt return of the stock. At the same time, blood cultures were performed on patients at risk, but no positivity was reported on other patients except the above-mentioned sample.

## Discussion

Here, we report on a multicentre outbreak of bloodstream infection caused by *C. viswanathii*, a yeast widely used in bioprocessing (e.g. oil degradation) and infrequently associated with invasive human infections [4]. Through coordinated epidemiological and microbiological investigations, the outbreak was ultimately linked to multiple contaminated batches of milrinone, a medicinal product purchased from an Indian company. In this context, WGS was instrumental in supporting the clonal relatedness between clinical and drug-derived isolates, providing robust genomic evidence to confirm the outbreak source.

The rarity of *C. viswanathii* and the diagnostic challenges associated with its identification probably contributed to delayed recognition of this outbreak. Initial misidentification as *C. tropicalis* in one centre is consistent with previous reports and underscores the limitations of some MALDI-TOF mass spectrometry systems in differentiating phylogenetically related species, particularly when reference spectral databases are incomplete or not regularly updated. Notably, the use of chromogenic media may aid in differential species identification and reduce the likelihood of misclassification as *C. tropicalis* [3]; nevertheless, colony pigmentation and macroscopic phenotypes can be heterogeneous and medium-dependent, overall accounting for a variable visual discrimination. These findings reinforce the importance of advanced mycological identification (e.g. amplicon sequencing of the ITS1/2 regions or the D1/D2 domains of the large-subunit rDNA) and routine susceptibility testing, particularly when uncommon species are isolated from sterile sites.

Clinical management of *C. viswanathii* infections remains challenging because of limited evidence and variable antifungal susceptibility profiles. Published reports describe heterogeneous MIC distributions, including reduced susceptibility to fluconazole in some isolates, which may necessitate alternative azole therapy or prolonged treatment courses [2,5].

This outbreak further adds to the growing body of evidence linking contaminated medicinal products to severe invasive fungal disease. Similar to previously reported outbreaks involving contaminated injectable drugs, the event described here demonstrates how contamination can result in multicentre dissemination, delayed detection and significant public health consequences [5,10,12]. The identification of milrinone as the common exposure was only possible through hypothesis-driven epidemiological reasoning informed by knowledge of the geographical distribution of *C. viswanathii*. Following notification to all participating centres and withdrawal of the implicated product, enhanced surveillance identified no additional cases after October 2025, supporting the effectiveness of the control measures implemented.

This study has several limitations. Firstly, case finding was laboratory-based and retrospective, so a degree of under-ascertainment cannot be excluded. Secondly, by design the case definition required exposure to milrinone, which precludes assessment of any *C. viswanathii* infection not linked to the drug. Thirdly, detailed individual clinical data, including comorbidities, antifungal treatment and outcomes, were outside the scope and governance of this emergency investigation and will be reported separately. Fourthly, exposure denominators were not available for all centres, precluding centre-level attack-rate estimation. Fifthly, WGS was performed only on a subset of isolates (31 of 40 clinical isolates and two of three drug isolates). Finally, an independent manufacturer-level root cause investigation was not accessible to the investigators.

## Conclusions

This multicentre outbreak of *C. viswanathii* candidaemia highlights the clinical and public health risks associated with contamination of medicinal products and the challenges posed by rare fungal pathogens. Accurate species identification, antifungal susceptibility testing and awareness among clinicians were critical to the identification and management of this outbreak. The investigation underscores the need for robust pharmacovigilance, strict adherence to good manufacturing practice by manufacturers, and effective inter-centre communication to prevent, detect and control similar events. Rare pathogens such as *C. viswanathii* should prompt consideration of exogenous sources, including contaminated pharmaceuticals, especially when infections occur across multiple centres without prior evidence of local transmission.

## Data Availability

Raw Illumina reads and draft genomes of *C. viswanathii* isolates included in this study have been deposited in NCBI databases under BioProject nos. PRJNA1372482, PRJEB108827 and PRJEB104646.

## References

[1] Viswanathan R Randhawa HS. Candida viswanathii sp. novo isolated from a case of meningitis. Science and Culture (Calcutta). 1959;25:86-7.

[2] Morvil N Wong HM. Recurrent knee septic arthritis and osteomyelitis due to Candida viswanathii: a case report and literature review. BMC Infect Dis. 2025;25(1):853. 10.1186/s12879-025-11188-8 40596890 PMC12217292

[3] Vrenna G Fox V Cortazzo V Raimondi S Cristiano M Foglietta G When conventional methods fail: first detection of a Candida viswanathii outbreak in Europe in a pediatric hospital revealed by whole genome sequencing and FT-IR spectroscopy. Microorganisms. 2025;13(12):2698. 10.3390/microorganisms13122698 41471902 PMC12734905

[4] Francisco EC Caceres DH Brunelli JGP Garcia-Effron G Arastehfar A Ribeiro FC An update on clinically relevant, rare, and emerging *Candida* and Saccharomycotina yeasts that have been recently reclassified from *Candida.* Clin Microbiol Rev. 2025;38(4):e0006423. 10.1128/cmr.00064-23 40900145 PMC12697193

[5] Shankarnarayan SA Rudramurthy SM Chakrabarti A Shaw D Paul S Sethuraman N Molecular typing and antifungal susceptibility of Candida viswanathii, India. Emerg Infect Dis. 2018;24(10):1956-8. 10.3201/eid2410.180801 30226183 PMC6154138

[6] Ciurea CN Kosovski IB Mare AD Toma F Pintea-Simon IA Man A. Candida and candidiasis-opportunism versus pathogenicity: a review of the virulence traits. Microorganisms. 2020;8(6):857. 10.3390/microorganisms8060857 32517179 PMC7355540

[7] Ahmed MAEE Abbas HS Kotakonda M. Fungal diseases caused by serious contamination of pharmaceuticals and medical devices, and rapid fungal detection using nano-diagnostic tools: a critical review. Curr Microbiol. 2023;81(1):10. 10.1007/s00284-023-03506-7 37978091 PMC10656328

[8] Benedict K Richardson M Vallabhaneni S Jackson BR Chiller T. Emerging issues, challenges, and changing epidemiology of fungal disease outbreaks. Lancet Infect Dis. 2017;17(12):e403-11. 10.1016/S1473-3099(17)30443-7 28774697 PMC5712439

[9] Ahearn DG Stulting RD. Moulds associated with contaminated ocular and injectable drugs: FDA recalls, epidemiology considerations, drug shortages, and aseptic processing. Med Mycol. 2018;56(4):389-94. 10.1093/mmy/myx070 29420758

[10] Ahearn DG Doyle Stulting R. Fungi associated with drug recalls and rare disease outbreaks. J Ind Microbiol Biotechnol. 2014;41(11):1591-7. 10.1007/s10295-014-1503-7 25173741

[11] Mugoyela V Mwambete KD. Microbial contamination of nonsterile pharmaceuticals in public hospital settings. Ther Clin Risk Manag. 2010;6:443-8. 10.2147/TCRM.S12253 20957135 PMC2952482

[12] Vijayakumar R Saleh Al-Aboody M Sandle T. A review of melanized (black) fungal contamination in pharmaceutical products--incidence, drug recall and control measures. J Appl Microbiol. 2016;120(4):831-41. 10.1111/jam.12888 26119714

[13] Tavares M Kozak M Balola A Sá-Correia I. Burkholderia cepacia complex bacteria: a feared contamination risk in water-based pharmaceutical products. Clin Microbiol Rev. 2020;33(3):e00139-19. 10.1128/CMR.00139-19 32295766 PMC7194853

[14] Tyski S Burza M Laudy AE. Microbiological contamination of medicinal products -is it a significant problem? Pharmaceuticals (Basel). 2025;18(7):946. 10.3390/ph18070946 40732236 PMC12300887

[15] Francis GS Bartos JA Adatya S. Inotropes. J Am Coll Cardiol. 2014;63(20):2069-78. 10.1016/j.jacc.2014.01.016 24530672

[16] Marino BS Tabbutt S MacLaren G Hazinski MF Adatia I Atkins DL Cardiopulmonary resuscitation in infants and children with cardiac disease: a scientific statement from the American Heart Association. Circulation. 2018;137(22):e691-782. 10.1161/CIR.0000000000000524 29685887

[17] Harris PA Taylor R Thielke R Payne J Gonzalez N Conde JG. Research electronic data capture (REDCap)--a metadata-driven methodology and workflow process for providing translational research informatics support. J Biomed Inform. 2009;42(2):377-81. 10.1016/j.jbi.2008.08.010 18929686 PMC2700030

[18] Harris PA Taylor R Minor BL Elliott V Fernandez M O’Neal L The REDCap consortium: Building an international community of software platform partners. J Biomed Inform. 2019;95:103208. 10.1016/j.jbi.2019.103208 31078660 PMC7254481

[19] Codda G Willison E Magnasco L Morici P Giacobbe DR Mencacci A In vivo evolution to echinocandin resistance and increasing clonal heterogeneity in *Candida auris* during a difficult-to-control hospital outbreak, Italy, 2019 to 2022. Euro Surveill. 2023;28(14):2300161. 10.2807/1560-7917.ES.2023.28.14.2300161 37022211 PMC10283462

[20] Li H Durbin R. Fast and accurate long-read alignment with Burrows-Wheeler transform. Bioinformatics. 2010;26(5):589-95. 10.1093/bioinformatics/btp698 20080505 PMC2828108

[21] Minh BQ Schmidt HA Chernomor O Schrempf D Woodhams MD von Haeseler A IQ-TREE 2: new models and efficient methods for phylogenetic inference in the genomic Era. Mol Biol Evol. 2020;37(5):1530-4. 10.1093/molbev/msaa015 32011700 PMC7182206

